# Mechanical trauma in children and adolescents in Berlin

**DOI:** 10.1007/s12024-024-00814-7

**Published:** 2024-04-16

**Authors:** Christine Eimer, Claas Buschmann, Jonas Deeken, Thoralf Kerner

**Affiliations:** 1 Department of Anaesthesiology and Intensive Care Medicine, University Hospital Schleswig-Holstein, Campus Kiel, Arnold-Heller-Str. 3, Kiel, Germany; 2 Institute of Legal Medicine, University Hospital Schleswig-Holstein, Kiel / Lübeck, Germany; 3 Asklepios Medical School GmbH, Lohmühlenstraße 5, Haus P, Hamburg, 20099 Germany; 4 Department for Anesthesiology, Intensive Care Medicine, Emergency Medicine, Pain Therapy, Asklepios Medical Centre, Harburg, Germany

**Keywords:** Trauma mechanism, Polytrauma, Traumatic brain injury, Pediatric trauma, Pediatric suicide

## Abstract

Management of severe pediatric trauma remains challenging. Injury patterns vary according to patient age and trauma mechanism. This study analyzes trauma mechanisms in deceased pediatric patients. Fatal pediatric trauma cases aged 0–18 years who underwent forensic autopsy in the Federal State of Berlin, Germany, between 2008 until 2018 were enrolled in this retrospective study. Autopsy protocols were analyzed regarding demographic characteristics, trauma mechanisms, injury patterns, resuscitation measures, survival times as well as place, and cause of death. 71 patients (73% male) were included. Traffic accidents (40%) were the leading cause of trauma, followed by falls from height > 3 m (32%), railway accidents (13%), third party violence (11%) and other causes (4%). While children under 14 years of age died mostly due to traumatic brain injury (59%), polytrauma was the leading cause of death in patients > 14 years (55%). Other causes of death were hemorrhage (9%), thoracic trauma (1%) or other (10%). A suicidal background was proven in 24%. In the age group of > 14 years, 40% of all mortalities were suicides. Cardiopulmonary resuscitation was carried out in 39% of all patients. 42% of the patients died at the scene. Children between 0 and 14 years of age died most frequently from traumatic brain injury. In adolescents between 14 and 18 years of age, polytrauma was mostly the cause of death with a high coincidence of suicidal deaths. The frequency of fatal traffic accidents and suicides shows the need to improve accident and suicide prevention for children and adolescents.

## Background

Management of pediatric trauma remains a challenge in both pre-hospital settings and emergency departments. While minor injuries among pediatric patients are frequent, life-threatening injuries in children and adolescents are rare: Buschmann et al. reported an incidence of major pediatric trauma as defined by an Injury Severity Score equal or greater than 16 points in 3% of all admissions to 103 German trauma centers [1]. In a more recent publication including 47.915 patients, the proportion of severely injured pediatric patients was 7% [2]. The term “mechanical trauma” describes injuries caused by blunt force, violent acts, acceleration, and deceleration trauma. Previous studies have shown that children are at certain risk of domestic violence, unintended accidents, and severe traffic accidents [3, 4]. Further studies on this topic have repeatedly revealed differences in injury mechanisms between pediatric and adult patients, which may also impact mortality [5, 6]. Compared to adults, pediatric trauma is rare, but usually survived, which may also be related to the fact that adults suffer far more frequently from comorbidities such as intoxications and pre-existing diseases [7].

The aim of this retrospective study was to gain further insights into trauma mechanisms among pediatric trauma mortalities, to draw attention to characteristics and to raise awareness of specific injury patterns.

## Methods

This retrospective analysis includes deceased pediatric trauma patients aged 0–18 years who underwent forensic autopsy in the Federal State of Berlin, Germany, between 2008 and 2018. All autopsies were performed in the Institute for Legal Medicine of the Charité - Universitätsmedizin Berlin. Cases were excluded if death occurred due to hanging, suffocation, drowning, burns, intoxications, or death after life-limiting illness.

Patients’ age, gender, type of trauma, trauma mechanism, time of event, resuscitation measures, survival time, place of death, and cause of death were analyzed. The study group was divided into three age categories (0–6 years; 7–13 years, 14–18 years) to provide granular data on age specific characteristics, gender distributions, accident timing, death mechanisms and causes of death. The type of trauma was differentiated into blunt and penetrating trauma. For better comparability of accident timing, four-hour slots were chosen, dividing the day into six Sect. (0:00–3:59 am; 4:00–7:59 am; 8:00–11:59 am; 0:00–3:59 pm; 4:00–7:59 pm; 8:00–11:59 pm). The place of death was categorized as follows: At the scene, during transport, in the emergency room, in the operating room and in the intensive care unit. The trauma mechanisms were labeled as fall from height > 3 m, railway accidents, third party violence, traffic accidents as pedestrian, two-wheel rider or car driver/passenger. Third party violence was defined as child assault or child abuse.

The causes of death in traffic accidents and falls from height > 3 m were further reviewed regarding injury pattern, patients’ age, and suicidal background. Causes of death were categorized as polytrauma, traumatic brain injury, hemorrhage and thoracic trauma as indicated in the autopsy protocols. Injury patterns were divided by injured body parts: head/neck, face, thorax, abdomen, extremity, and great vessel injuries and additionally split into the age groups mentioned above. Suicides were identified according to police investigations. Suicides were evaluated regarding gender, patient age, frequency, and trauma mechanism. Statistical analysis was performed with Graph Pad Software, Prism Version 10.1.0. An ethical approval was not required due to the retrospective evaluation of anonymized data sets. The study was performed in accordance with the 1964 Helsinki declaration.

## Results

This study includes 71 pediatric patients who died due to mechanical trauma in Berlin, Germany, between 2008 and 2018. Patients´ demographics are shown in Table 1.

**Table 1:** Patients’ demographics

Gender, n (%)	
Male	52 (73%)
Female	19 (27%)
***Age, n (%)***	
0–6 years	19 (27%)
7–13 years	10 (14%)
14–18 years	42 (59%)
***Type of injury, n (%)***	
Blunt	65 (92%)
Penetrating	3 (4%)
Both	3 (4%)
***Mechanism of injury, n (%)***	
Traffic accident	28 (40%)
- pedestrian	14
- two-wheel	5
- car	9
Fall from height > 3 m	23 (32%)
Railway accident	9 (13%)
Third party Violence	8 (11%)
Others	3 (4%)

**Fig. 1:**
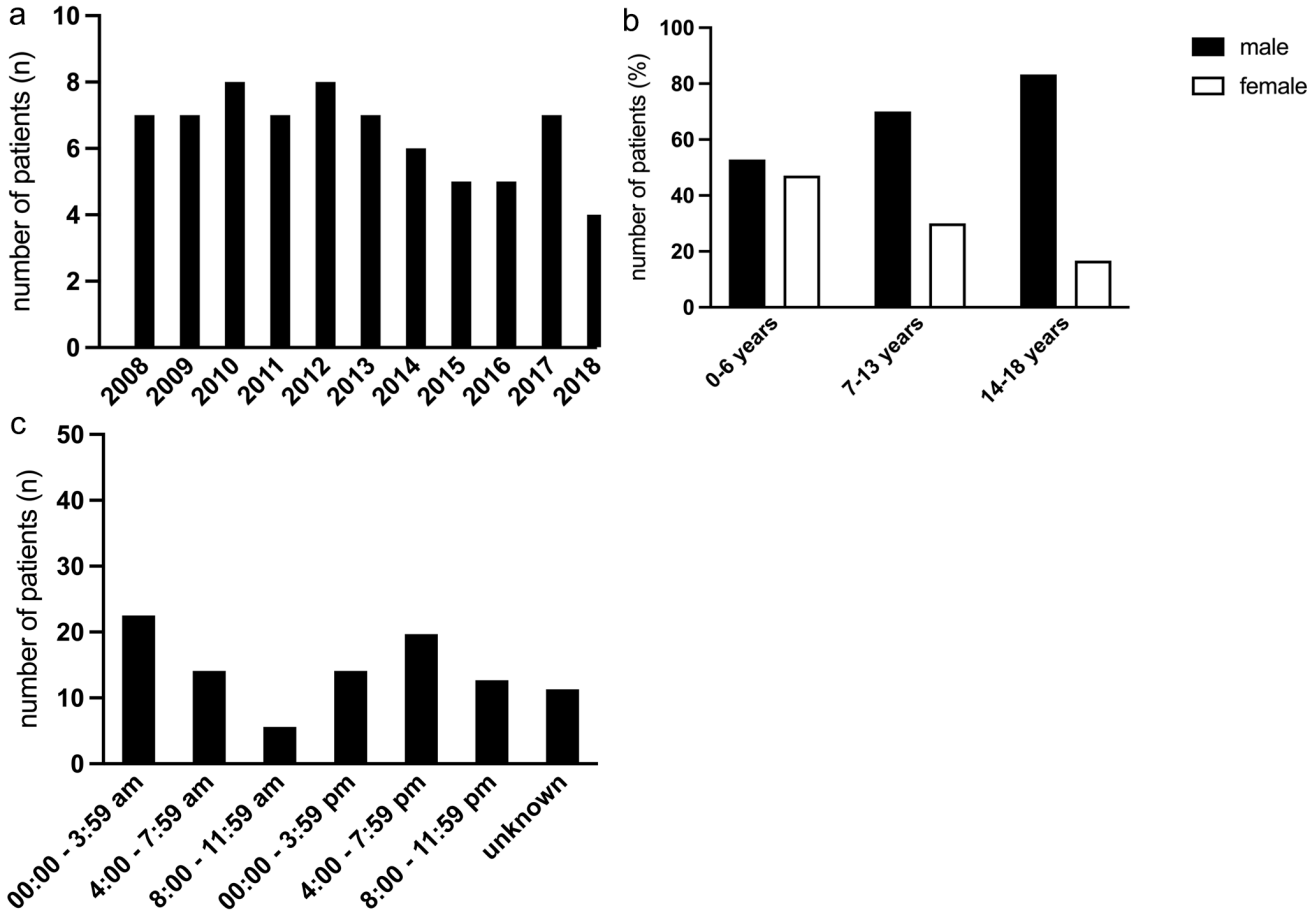
General characteristics. (**a**) Frequency of death per year (**b**) Representation of gender distribution in relation to age and number of cases. (**c**) Illustration of events in relation to the time of day

The distribution of injury mechanism depending on patients’ age is shown in Table 2. Figure 1a and b indicate the frequency of fatal trauma per year and represent the distribution of gender in relation to age. Most of the accidents happened between 0:00–4:00 am (22.5%) and 4:00–7:59 pm (19.7%) (Fig. 1c).

**Table 2:** Mechanism of injury by age group

Age group	Traffic accidents	Falls from height > 3 m	Railway accidents	Third party Violence	Others	
0–6 years	6	8	1	3	1	**19 (26.7%)**
7–13 years	4	1	1	3	1	**10 (14.1%)**
14–18 years	18	14	7	1	2	**42 (59.2%)**

**Fig. 2:**
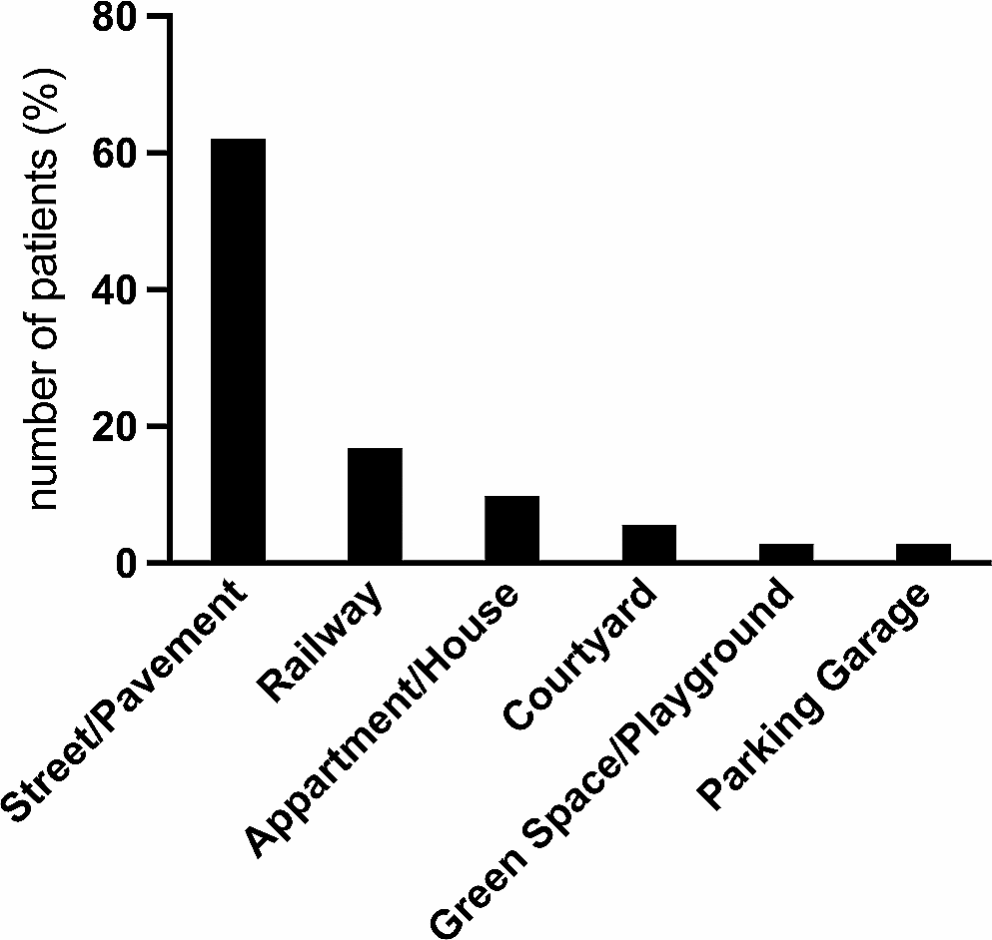
Scene characteristics. Illustrates the frequency of scene characteristics

Cardiopulmonary resuscitation was performed in 28 (39%) patients at the scene, Fig. 2 illustrates scene characteristics. 30 (42%) died at the scene, 13 (18%) in the emergency department, 25 (37%) on the intensive care unit, 2 (3%) in the operation room and 1 (1%) after discharge from hospital. 53 (75%) of all victims died within 24 h of the event, 18 (25%) died in the further course of time.

**Fig. 3:**
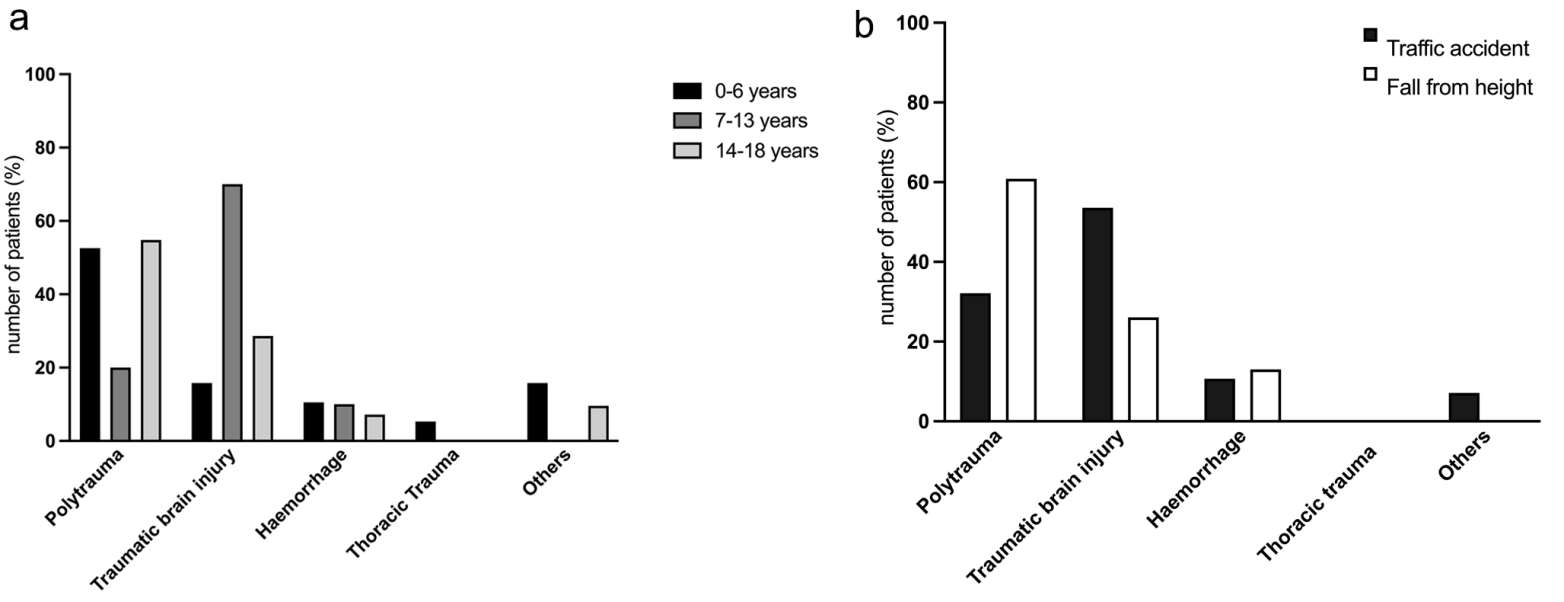
Causes of death. (**a**) Causes of death in relation to age (**b**) Illustration of causes of death in relation to trauma mechanism

The leading cause of death was traumatic brain injury in 29 patients (41%), followed by polytrauma in 28 cases (39%), hemorrhage in 6 cases (9%), chest trauma in one case (1%) and other causes in 7 patients (10%). Injury patterns in relation to patient age are illustrated in Table 3. Figure 3 illustrates causes of death.

**Table 3:** Injury pattern

	Head/Neck	Face	Thorax	Abdomen	Vessels	Extremities
0–6 years (*n* = 19)	17 (90%)	7 (37%)	10 (53%)	6 (32%)	1 (5%)	5 (26%)
7–13 years (*n* = 10)	9 (90%)	2 (20%)	3 (30%)	2 (20%)	1 (10%)	3 (30%)
14–18 years (*n* = 42)	34 (81%)	9 (21%)	28 (67%)	19 (45%)	8 (19%)	28 (67%)

### Suicide

**Fig. 4:**
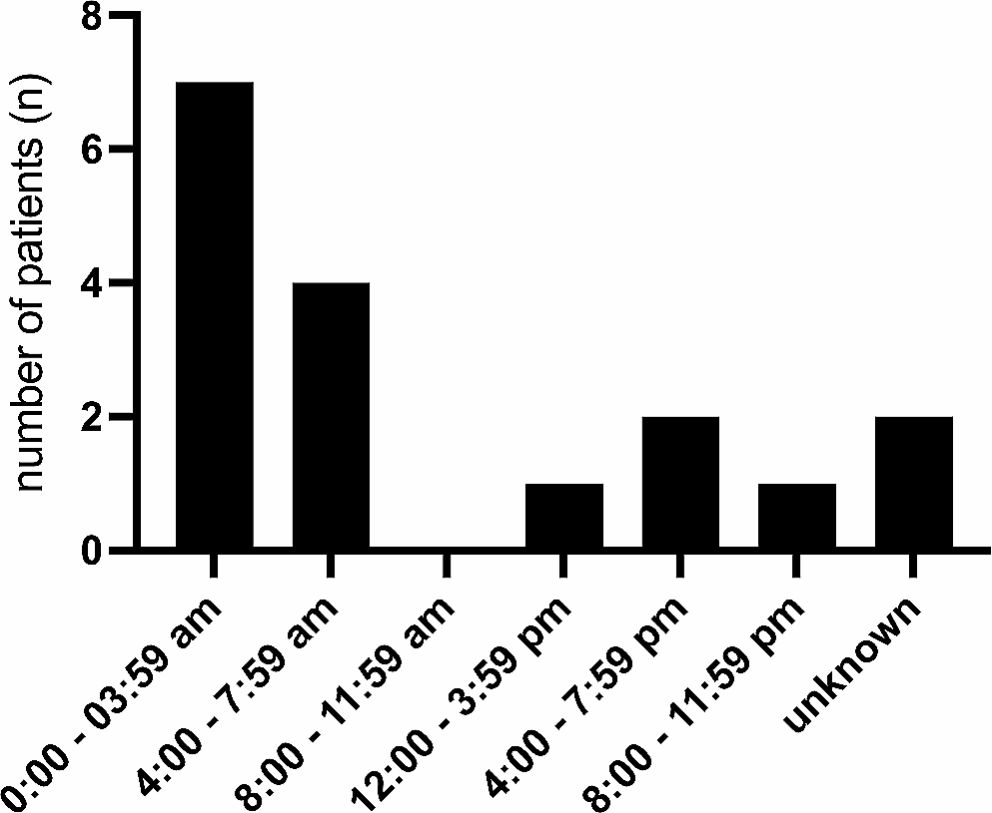
Suicide times. Illustration of suicides in relation to the time of the day

17 cases were suicides (13 male, 4 female). There were no suicides in children between 0 and 13 years of age, but 40% of all incidents in the 14–18 years old age group were committed with suicidal intent. The youngest suicide death case was 14 years with an increasing number of suicides until the age of 18 years. Most suicides occurred between 0:00 a.m. and 8:00 a.m. (Fig. 4) and were evenly distributed throughout the year (Jan-Mar = 5; Apr – Jun = 5; Jul – Sep = 3; Oct – Dec = 4). The most frequently chosen mechanism was fall from height > 3 m in 11 patients, 5 patients died on railway tracks and 1 patient died from a self-inflicted gunshot wound.

### Traffic accidents

**Fig. 5:**
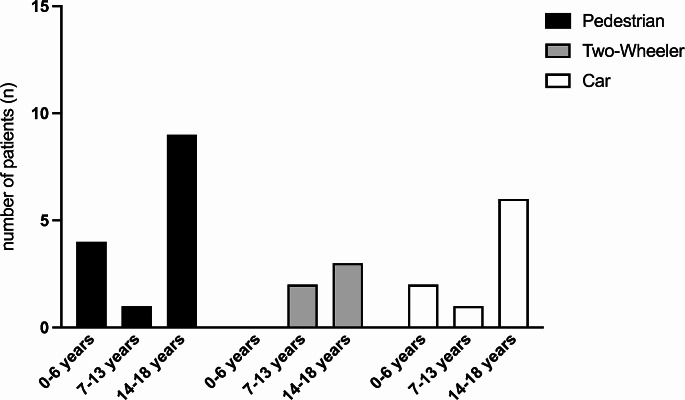
Distribution of age in relation to frequency and type of traffic accidents

A total of 28 (40%) patients died from traffic accidents. The highest proportion of deaths due to traffic accidents occurred as pedestrians (14/28), followed by car accidents and bicycles (resp. one case with a motorbike) (Fig. 5). There was a time peak between 4:00 p.m. and 7:59 p.m. for children up to 13 years of age; in the age group over 14 years of age, accidents occurred most frequently in nighttime hours between 0:00 a.m. and 3:59 a.m. The highest number of traffic accidents was documented between April and June.

### Fall from height > 3 m

Fall from height > 3 m resulted in death in 23 cases including 11 suicides. Falls from height > 3 m occurred in all age groups, with 1 case in the age group < 1 year as part of a violent crime, 7 cases in the age group 1–6 years old, 1 case in the age group 7–13 years and 14 cases in the 14–8-year-old age group. The most common cause of death in patients with falls from height > 3 m was a polytrauma in 61% followed by traumatic brain injury in 26% and fatal hemorrhage in 13%.

### Railway fatalities

Railway fatalities occurred in 9 cases including 5 suicides. Two deaths were documented for the age between 0 and 13 years and 7 railway deaths occurred in the age group between 14 and 18 years.

### Third party violence

Third party violence was the cause of death in 8 patients (11%), including punches, falls from height > 3 m and sharp force. Third party violence was the cause of death in 7 (24%) of all patients aged 13 and younger. Three infants (< 2 years of age) died from third party violence, two of them died of a traumatic brain injury. In the age group 14–18 years, third party violence occurred less frequent and was the cause of death in only one case (2%).

A total of 3 cases could not be assigned to a category, including a fallen cupboard on a 1-year-old child, a fall from the subway and a jump out of a car.

## Discussion

Trauma contributes significantly to mortality in childhood [8]. The aim of this study was to analyze injury patterns in relation to trauma mechanism and subsequent causes of death. Although mechanisms of injury vary between age groups, vehicle and fall-related injuries were most common mechanisms in all age groups. A suicidal motivation was identified in 17 out of 71 patients, and 40% of all fatalities between 14 and 18 years committed suicide. Most common cause of death in children up to the age of 13 was traumatic brain injury (59%) while adolescents died more often from polytrauma (55%). Overall, 75% died within 24 h of the event, which is in line to other studies reporting similar rates [9].

Various studies worldwide have shown that traffic accidents are the most common cause of trauma and the leading cause of death in children [3, 10–12]. In our study, 40% deaths were related to traffic accidents. Comparable to other studies, we observed lower percentages of traffic accidents in the lower age groups, however, percentages increased significantly in older patients [12]. Most fatal traffic accidents in 14–18 years old victims occurred as pedestrians and two-wheelers, which can be explained by the increasing participation in road traffic. The predominant victims in this age group were male. Age-dependent behavioral changes and an increasing willingness to take risks – especially in male children – may also contribute to the frequency of severe trauma in adolescents. Traumatic brain injury was the leading cause of death in fatal traffic accidents. Previous studies in adults revealed that pedestrians – followed by cyclists – are the most vulnerable group among traffic accident victims with the highest risk of traumatic brain injury [13, 14]. Children have several anatomical differences that may predispose them to injury such as a more vulnerable cranial vault due to thinner bones and a larger head-to-torso ratio [6]. Therefore, children might be at even greater risk of injury than adults who experience a comparable trauma. In the present study, 5 patients died due to accidents with two-wheels. Cycling does not require mandatory helmet use in Germany, which may contribute to an increased risk of head injury after fall. However, strategic measures such as infrastructural improvements in roads, speed limits or autonomous braking systems may certainly also have great influence on children’s’ safety in traffic.

As already reported in previous studies, falls from height > 3 m account for an important proportion of pediatric trauma and childhood mortality [3, 4]. In the presented study, falls from height > 3 m were the most common cause of trauma in children aged 0–6 years. Regarding our data, 22 (31%) patients died from falls from height > 3 m due to unintended accidents, suicides and third-party violence. Height and speed of the fall determine the severity of the injury, however, due to numerous determinants, correlations between height and corresponding injury pattern remain challenging [15]. In our study, drop height was only categorized in “ground level fall” and “fall from height > 3 m”. Nevertheless, the comparison of injury pattern in deceased patients with fatal traffic accidents and falls from height > 3 m revealed major differences.

The injury pattern in falls from height > 3 m revealed severe injuries of head, neck, thorax, abdomen, vessels and extremities resulting in severe polytrauma being the most common cause of death. Nevertheless, injury pattern may differ depending on age, fall height and victim´s intention to fall [16]. Due to the limited number of patients, the variety of patient age and both suicidal and accidental falls from height > 3 m, interpretation of our findings is limited. Further research is necessary to gain more insights in pediatric injury pattern related to accidental and suicidal falls. In our data, three adolescents died due to a fall from height > 3 m between 0 and 5 a.m. without a suicidal background being proven. Considering the frequency of suicides in this age group and the typical event times during night hours, suicides would still be conceivable.

5 out of 9 patients who died due to traumatic railway injury committed suicide, and 7 out of 9 patients were 14–18 years old. Fatal self-inflicted injury is one of the leading causes of death among young people. In our study, 17 patients committed suicide with 77% being male, age ranged from 14 to 18 years, with a mean age of 16 years. The results correspond to other studies, showing increased suicide rates during puberty [17] and among male adolescents [18]. Male suicidents tend to choose rather “violent” suicide methods with a higher risk of death, which might explain this gender discrepancy. Due to the focus on “trauma” in this study, causes of death such as “intoxication” which might be preferred by female suicidents were excluded from our analysis [18].

Eight patients were killed by third party violence, 7 of whom were younger than 13 years. Surprisingly, third party violent acts were responsible for 24% of fatal trauma between 0 and 13 years. Recognizing violence against children and its prevention remain great challenges. Global evidence reveals that the prevalence is highly underestimated [19–21]. Most of the children in our study were beaten to death, one died from a violence-related fall from height > 3 m, one 8-year-old girl bled to death due to a cut through the carotid artery and one 17-year-old boy died from a cardiac stab wound.

A relevant proportion of traumatic deaths occurred on-scene, i.e., before arriving at hospital (42%). This highlights once again that the pre-hospital setting remains a hot spot of trauma mortality [7].

### Limitations

Data analysis regarding potential preventable trauma deaths was not performed in the presented study. Some cases in the age group of 0–6 years suggest that a neglect of adequate supervision may have contributed to fatal accidents, this should be further analysed in follow-up studies. Only autopsy reports of deceased children and adolescents were analyzed. Conclusions about non-fatal injury patterns and the incidence of non-fatal pediatric trauma events cannot be derived. Antunez et al. reported that perimortem full body computed tomography may be beneficial in identifying causes of death in children with severe trauma, futures studies should therefore also focus on radiological imaging [22]. autopsy rates are low in Germany, and even pediatric trauma fatalities do not always undergo forensic autopsy. Thus, there might be a bias between incidence of fatal pediatric trauma in a certain area and performed forensic autopsies in trauma death cases in that area. Besides, there is a second institution in Berlin which performs forensic autopsies, the State Institute of Forensic Medicine. Autopsy reports from this institution were not included in our study but should also account for about 70 pediatric death cases during the study period.

## Conclusion

Traffic accidents and falls from height > 3 m were the most common trauma mechanisms leading to death in children and adolescents. Children were affected by violence in a relevant proportion of cases. A suicidal background was very common within the group of the 14–18 years old. Injury pattern varied according to age and trauma mechanism, nevertheless, most common cause of death in children up to the age of 13 was traumatic brain injury, adolescents died more often from polytrauma.

## Key points

1. The majority of patients in this study were male.
2. Children under 14 years of age died mostly due to traumatic brain injury.
3. Polytrauma was the leading cause of death in patients > 14 years of age.
4. 24% of trauma fatalities were suicides.
5. 24% deaths under 13 years were due to third party violence.
6. Death occurred within 24 h after trauma in 75% of all cases.
